# Concealed Enterovesical Fistula Associated with Forgotten Intra-Abdominal Haemostat and Intravesical Towel

**DOI:** 10.1155/2014/723592

**Published:** 2014-04-14

**Authors:** Ademola Alabi Popoola, J. O. Bello, G. G. Ezeoke, K. T. Adeshina, A. Fadimu

**Affiliations:** ^1^Urology Unit, Department of Surgery, University of Ilorin Teaching Hospital, University of Ilorin, P.O. Box 4718, Ilorin 24001, Kwara State, Nigeria; ^2^Department of Obstetrics and Gynaecology, University of Ilorin Teaching Hospital, University of Ilorin, P.O. Box 4718, Ilorin 24001, Kwara State, Nigeria

## Abstract

*Introduction.* Enterovesical fistula is rare and is often caused by bowel inflammatory diseases and tumours in the urinary bladder or the intestine with local infiltration of bowel or bladder, respectively. The fistula usually presents with lower urinary tract symptoms, pneumaturia, and faecaluria or with food particles in the urine. Intra-abdominal retained surgical foreign bodies have also been reported as causes. * Case Presentation.* A case of atypical presentation in a woman with enterovesical fistula following abdominal hysterectomy. Investigations confirmed the presence of surgical towel in the urinary bladder and a pair of artery forceps in the abdomen. The towel was removed at cystoscopy after which she presented with food particles in the urine. She later had laparatomy to remove the haemostat and to repair the fistula. * Discussion.* A typical presentation of enterovesical fistula delayed the diagnosis and treatment in this patient. * Conclusion.* Managing patients with recurrent urinary tract infection after abdominal operation should include appropriate imaging of the abdomen with emphasis on pelvic organs. Also, surgical operation should always be given the best shot the first time and strict operation room standards and guidelines should always be followed.

## 1. Introduction

Enterovesical fistula (EVF) is an abnormal communication between the intestine and the urinary bladder. It is rare and studies have reported an incidence of less than 4 cases per year [1, 2]. Enterovesical fistulae often result from local infiltration of the urinary bladder by intestinal tumours resulting in a communication between these two luminal organs [3]. However, there have been reports of reversed scenarios whereby the fistula followed invasion of contiguous loops of bowel by squamous cell carcinoma of the urinary bladder [4]. Apart from tumours, bowel inflammatory diseases such as Crohn's disease also rank high in the etiology of EVF [5]. Urethral catheterization of compromised urinary bladders after external beam radiotherapy for pelvic tumours has also been implicated in the aetiology [6]. Spontaneous occurrence of EVF attributable to no specific cause has also been reported [7]. EVF usually presents with symptoms such as the presence of food particles in the urine, lower urinary tract symptoms, pneumaturia, and faecaluria. Alapont Pérez et al. reported that 78% of their patients presented with pneumaturia and faecaluria [8]; it is therefore uncommon for EVF to remain occult betraying with no classical symptoms or signs.

Retained surgical foreign bodies (RSFB) following operative procedures have been reported [9–11]. The exact incidence rate may be difficult to ascertain for reasons which may include but are not limited to the fear of litigation [12]. There are various complications associated with RSFB which range from abdominal pains [10] to death [9]. Gossypiboma (retained gauze) usually presents with symptoms such as abdominal pain, swelling, or signs of occult infections [13]

Intra-abdominal foreign bodies have been associated with erosion into luminal or hollow structures creating different forms of internal and external fistulae with various presentations depending on the structures involved. There have been reports of aortoenteric fistulae from RSFB manifesting as gastrointestinal hemorrhages [14]. Entero- or colocutaneous fistulae have also resulted from RSFB [15]. We report a case of EVF associated with RSFB with unusual presentation.

## 2. Case Presentation

A 48-year-old obese woman was referred to our unit following an ultrasound finding of an echogenic mass in the urinary bladder while investigating her for a 10-month history of recurrent lower urinary symptoms. Her symptoms started a few weeks after she had abdominal hysterectomy. This was her third intra-abdominal operation. The record of the hysterectomy showed that it was difficult and prolonged arising from extensive intra-abdominal adhesions. The procedure lasted for several hours necessitating the change of scrub nurses and the invitation of a senior gynaecologist. The postoperative period was turbulent as the patient developed postoperative sepsis and surgical site wound infection and, subsequently, superficial wound dehiscence. She was discharged after secondary suturing of the dehisced wound and total hospital stay was one month. After her discharge, she continued to have a gamut of complaints including lower abdominal pain and irritative lower urinary tract symptoms. There was no associated pneumaturia, faecaluria, hematuria, necroturia, or leakage of urine per rectum. Serial urine cultures yielded different coliforms and her lower urinary tract symptoms responded fleetingly to antibiotics based on urine culture sensitivity pattern each time.

**Figure 1:**
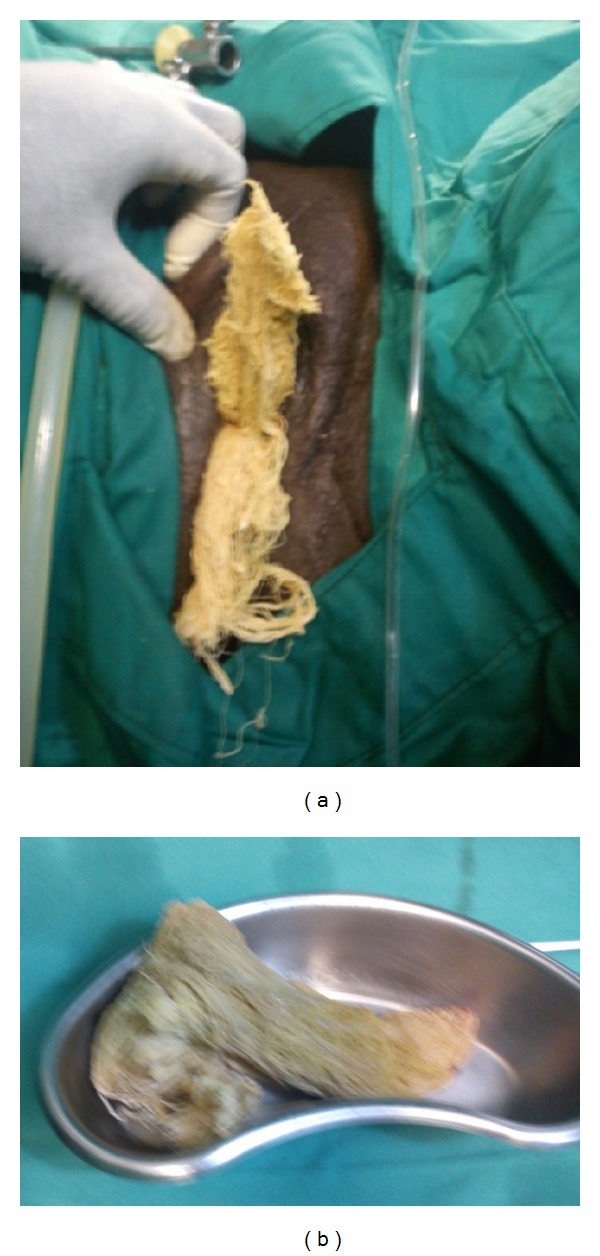
Gauze been pulled out through the urethral meatus and gauze after it was retrieved.

**Figure 2:**
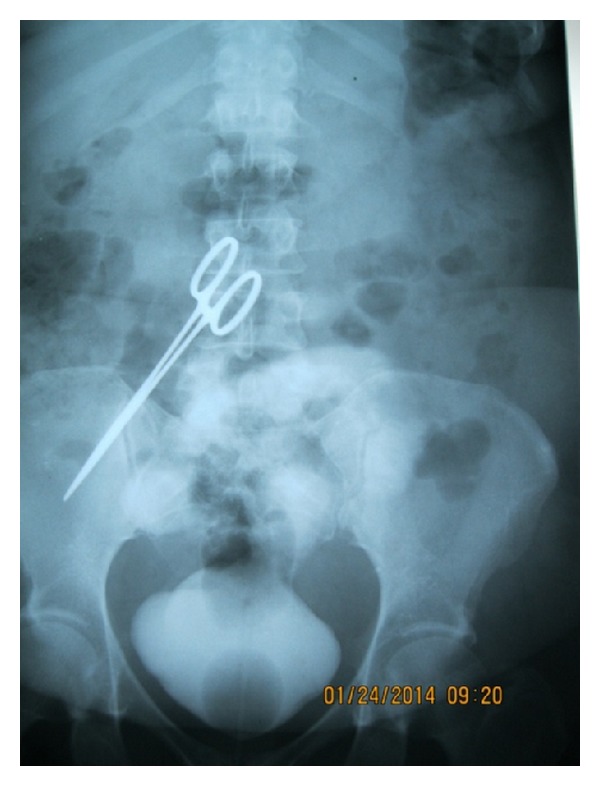
Cystogram showing contrast extravasation into the abdomen and a pair of artery forceps lying in abdominopelvic region.

Abdominal ultrasound scan done revealed an echogenic mass in the urinary bladder and urethrocystoscopy revealed a surgical towel in the bladder. A part of the towel was pulled out of the bladder by a grasper through the urethral meatus and then held by a pair of forceps. It was then pulled out gradually while being cut in bits and pieces were retrieved piecemeal endoscopically using a grasper and cut into bits and pieces with a pair of scissors as it was gradually retrieved (Figures 1(a) and 1(b)). A size 18 Fr urethral catheter was left in situ after endoscopy but it got blocked frequently from particles draining through it. Closer look at these revealed that these were food particles leading to the suspicion of the presence of an enterovesical fistula. A cystogram subsequently confirmed the diagnosis of enterovesical fistula. In addition, the cystogram revealed a surprise finding of a pair of haemostats which was seen lying in the abdominal cavity (Figure 2).

The patient had laparatomy during which the haemostat was found buried in adhesions with small bowel looping around it. It was removed and a piece of degenerating surgical textile material was found between the jaws of the haemostat. With little manipulation, the jaw of the haemostat got broken. A fistulous connection between the ileum and the dome of the urinary bladder was also found. The segment of the small bowel contiguous to the fistula was resected and sent for histology, and intestinal continuity was restored through an end to end anastomosis. The bladder defect was closed in two layers. The patient did well postoperatively and was discharged after about 10 days on admission. She has since been lost to follow-up.

## 3. Discussion

Typically, patients with EVF will present with faecaluria, pneumaturia, dysuria, hematuria, and chronic abdominal pain in hypogastric and left iliac regions [16]. However, the patient in this report did not have these classical features until the surgical towel in the urinary bladder was discovered and removed at cystoscopy. The impacted intravesical gauze was probably blocking the fistulous connection between the intestines and the urinary bladder, thereby concealing the presence of the fistula. This atypical presentation concealed the presence of the EVF, delayed the diagnosis, and prolonged the patient's predicament. The recurrent UTI was due to contamination of the urinary bladder with content from the small intestines seeping through the abdominal textile. It was therefore not surprising that the UTI was recurrent with urine culture yielding different coliforms with fleeting responses to several culture-specific antibiotic treatments. Recurrent UTI in a patient who had recently had abdominopelvic operation should always raise the possibility of an abnormal communication with urinary tract.

The simultaneous finding of intra-abdominal haemostat and intravesical surgical textile is quite rare. The explanation for the occurrence requires some deep thoughts and analysis. There are several reports of transmural migration of RSFB through several luminal intra-abdominal organs with various consequences [8, 9, 17]. However, to our knowledge, RSFB causing EVF has not been reported. We suspected that the artery forceps was used to tag the tail of abdominal pack and possibly, instead of being kept outside the abdominal cavity, it was not or in the heat of the difficult operation it got thrown into the abdominal cavity but not retrieved at the end of the operation. Hence it was forgotten together with the towel in the abdominal cavity. The towel probably eroded through the urinary bladder wall or possibly still there could have been a rent in the bladder wall created in the heat of the difficult operation while separating the adhesions. The tail of the towel could have been digested by the intra-abdominal enzymes, therefore detaching it from the haemostat. It is of note that degenerating textile was noticed between the jaws of the haemostat. A suggestion of a longer tail for abdominal packs will ensure that artery forceps tagging abdominal towels are always kept outside the abdominal cavity making them more unlikely to be forgotten. The formation of a communication between the urinary bladder with the intestine could be explained by the migration of the retained sponge into bowel as a result of inflammation in the intestinal wall that evolves to necrosis [18].

In this case, it was obvious that the operation was a difficult one which lasted for several hours, requiring the invitation of more experienced hands and a change of guards by the perioperative nurses. Such a scenario has at least three risk factors described by Stawicki et al. [19] in their comprehensive review of risks and preventive strategies of retained surgical foreign bodies. These risk factors are (i) involvement of more than one surgical team, (ii) prolonged surgical procedures, and (iii) complex surgical procedures. Proper preoperative planning usually will dictate that the more experienced hands are to be present at the beginning of an anticipated difficult operation. Another laparatomy in a patient who has had three previous ones should have suggested a difficult operation [20]. Having the more experienced hands at the beginning of the operation would probably have reduced the operation time and the need to have a change of scrub nurses. It cannot be overemphasized that perioperative nurses should always pay meticulous attention to instrument and material counts, especially when there is a prolonged procedure which may require additional instruments and the need for change of guards.

## 4. Conclusion

Surgical operation should always be given the best shot the first time and strict operation room standards and guidelines should always be followed.

Since RSFB cannot be completely avoided in surgical practice, it should be given considerations in the list of differential diagnoses in order to make early diagnosis and treatment. This is because it has been a cause of untold suffering, morbidity, and mortality, especially when diagnosed late. Also, whenever there is recurrent urinary tract infection especially in a postoperative patient, abdominopelvic ultrasound scan should be done to exclude the possibility of an intravesical foreign body.
